# Ferrofluid Impregnation Efficiency and Its Spatial Variability in Natural and Synthetic Porous Media: Implications for Magnetic Pore Fabric Studies

**DOI:** 10.1007/s11242-022-01809-0

**Published:** 2022-07-02

**Authors:** Michele Pugnetti, Yi Zhou, Andrea R. Biedermann

**Affiliations:** Institute of Geological Sciences, University of Bern, Baltzerstrasse 1+3, 3012 Bern, Switzerland

**Keywords:** Pore fabric characterization, Magnetic pore fabrics, Impregnation efficiency, Impregnation method

## Abstract

Magnetic pore fabrics (MPF) are an efficient way to characterize pore space anisotropy, i.e., the average pore shape and orientation. They are determined by impregnating rocks with ferrofluid and then measuring their magnetic anisotropy. Obtaining even impregnation of the entire pore space is key for reliable results, and a major challenge in MPF studies. Here, impregnation efficiency and its spatial variability are systematically tested for natural (wood, rock) and synthetic (gel) samples, using oil- and water-based ferrofluids, and comparing various impregnation methods: percolation, standard vacuum impregnation, flowthrough vacuum impregnation, immersion, diffusion, and diffusion assisted by magnetic forcing. Seemingly best impregnation was achieved by standard vacuum impregnation and oil-based ferrofluid (76%), and percolation (53%) on rock samples; however, sub-sampling revealed inhomogeneous distribution of the fluid within the samples. Flowthrough vacuum impregnation yielded slightly lower bulk impregnation efficiencies, but more homogeneous distribution of the fluid. Magnetically assisted diffusion led to faster impregnation in gel samples, but appeared to be hindered in rocks by particle aggregation. This suggests that processes other than the mechanical transport of nanoparticles in the pore space need to be taken into account, including potential interactions between the ferrofluid and rock, particle aggregation and filtering. Our results indicate that bulk measurements are not sufficient to assess impregnation efficiency. Since spatial variation of impregnation efficiency may affect MPF orientation, degree and shape, impregnation efficiency should be tested on sub-samples prior to MPF interpretation.

**Supplementary Information:**

The online version contains supplementary material available at 10.1007/s11242-022-01809-0.

## Article Highlights

- Even ferrofluid impregnation is a key prerequisite for reliable pore fabric characterization by magnetic methods
- Spatial variability of impregnation efficiency on sub-samples suggests that flow-through methods provide homogeneous impregnation
- Successful impregnation depends on pore size, wettability, existing pore fluid, impregnation method, and ferrofluid-sample interactions

**Supplementary Information:**

The online version contains supplementary material available at 10.1007/s11242-022-01809-0.

## Introduction

Magnetic pore fabrics (MPF), measured as anisotropy of magnetic susceptibility (AMS) in ferrofluid-impregnated samples, are a useful technique to investigate the average fabric of connected pores in rock samples (Benson et al. 2003; Hrouda et al. 2000; Louis et al. 2005; Parés et al. 2016; Pfleiderer and Halls 1990, 1993; Pfleiderer and Kissel 1994; Robion et al. 2014). Empirical relationships have been reported between the orientation of maximum susceptibility and the pore elongation direction (Hrouda et al. 2000; Pfleiderer and Halls, 1990, 1993), and between the degree of anisotropy and average pore shape (Jones et al. 2006; Pfleiderer and Halls, 1990, 1993). Additional correlations exist between MPFs and permeability anisotropy (Hailwood et al. 1999; Nabawy et al. 2009; Pfleiderer and Halls 1994). AMS methods are also commonly and successfully applied to characterize mineral alignment caused by deformation or transport (Borradaile and Henry 1997; Borradaile and Jackson 2010; Hrouda 1982). The term MPF will be used here to describe AMS measured on impregnated samples, whereas AMS will be used to describe measurements prior to impregnation. Previous studies identified time-efficiency and the potential to capture pores down to 10–20 nm, thus overcoming resolution limits inherent to imaging and tomography methods, as major advantages of MPFs compared to traditional pore fabric characterization methods (Almqvist et al. 2011; Humbert et al. 2012; Parés et al. 2016; Robion et al. 2014). At the same time, Almqvist et al. (2011) reported that the centre of their sample was not impregnated, and Robion et al. (2014) discussed potential limitations related to pore throats being smaller than pores, thus limiting the pores that are captured.

To measure MPFs, samples are impregnated with ferrofluids, i.e., colloidal suspensions of magnetite nanoparticles in water- or oil-based carrier liquids. Due to the high susceptibility of ferrofluid compared to main rock-forming minerals, the MPF is controlled by the distribution of the fluid in the pore space (Hrouda et al. 2000; Parés et al. 2016; Pfleiderer and Halls 1990). The interpretation of MPFs relies on the assumption that the entire accessible pore space is filled with ferrofluid evenly, and incomplete impregnation is expected to bias MPFs and derived pore space models. Therefore, impregnation efficiency is a key parameter in MPF studies, and successful impregnation methods need to provide high impregnation efficiency as well as homogeneous impregnation throughout the sample.

Most MPF studies have used standard vacuum impregnation, i.e., evacuating the pore space in a vacuum chamber for up to 48 h, and then supplying ferrofluid up to 24 h (Almqvist et al. 2011; Benson et al. 2003; Hrouda et al. 2000; Humbert et al. 2012; Nabawy et al. 2009; Parés et al. 2016; Pfleiderer and Halls 1990, 1993, 1994; Robion et al. 2014). Additionally, attempts have been made pumping ferrofluid through the sample by a pressure gradient (Pfleiderer and Halls 1990, we will refer to this method as 'flowthrough'), injecting the fluid under progressively higher pressure (Esteban et al. 2006), or using a specifically developed saturation cell (Hailwood et al. 1999). Impregnation efficiency, describing the percentage of pore space penetrated by ferrofluid, was evaluated by visual inspection of cut surfaces or thin sections (Pfleiderer and Halls, 1990, 1993), or by comparing the measured increase in weight or susceptibility during impregnation to independently measured porosity and ferrofluid properties (Almqvist et al. 2011; Benson et al. 2003; Nabawy et al. 2009; Parés et al. 2016; Robion et al. 2014). Mass and susceptibility changes quantify the amount of ferrofluid inside the sample and at the sample surface, but do not provide any information on the distribution of ferrofluid, nor potential preferred impregnation of certain pore sizes or shapes. Almqvist et al. (2011) used X-ray computed tomography to map the fluid within the pore space, and found that the fluid penetrated regions close to the sample surface, but did not reach the centre. To evaluate effects of incomplete ferrofluid penetration on the MPF orientation, Humbert et al. (2012) and Robion et al. (2014) measured three mutually perpendicular cores. For some studies, the impregnation efficiency can be estimated from the reported data: Mass-based impregnation efficiency estimates range between 39 – 154%, while susceptibility-based impregnation efficiency for the same samples is significantly lower, < 1–47% (Almqvist et al. 2011; Nabawy et al. 2009; Parés et al. 2016; Robion et al. 2014). Benson et al. (2003) reported ~ 90% impregnation, though their calculation used a fluid susceptibility significantly below that expected for their fluid and concentration; the expected susceptibility reduces the impregnation efficiency to 52%. These results highlight the need for improved impregnation protocols, and a better evaluation of the ferrofluid distribution.

One way to overcome incomplete impregnation is in-situ nanoparticle co-precipitation in the pore space, which allows injecting smaller particles with more favourable impregnation properties (Merk et al. 2014). Iron oxide particles of 22 nm ± 7 nm were observed, and this large range of particle sizes makes it hard to predict their magnetic properties. A promising alternative for future MPF studies could be fluids containing single molecule magnets (Murugesu et al. 2005; Soler et al. 2001; Sun et al. 1998). Here, we investigate potential improvements to impregnation methods. While vacuum impregnation is standard in MPF applications, pressure impregnation may reach higher impregnation efficiencies, though at the expense of potentially altering pore space (Esteban et al. 2006). Vacuum and pressure impregnation have been successfully used to impregnate wood, suggesting that additional pressure cycles increase impregnation efficiency up to > 94%, but lead to higher risk that pore space may be altered at each pressure cycle (Locs et al. 2008). Additionally, impregnation direction controls the impregnation efficiency, with higher impregnation reached when impregnating along wood fibres (Oka et al. 2002). This observation suggests that also the impregnation efficiency in rocks may depend on the orientation of the impregnation direction with respect to the pore fabric. Mazurek and Rufer (2018) describe an experimental setup to extract and characterize the pore water based on an adapted isotope diffusive exchange (AIDE) method. In their diffusion cell setup, the sample’s pore water is equilibrated with an external reservoir, to analyze its chemical composition. The method has been used to study transport properties and advection diffusion of radionuclides and tracers, and can be adapted to study the diffusion of ferrofluid into the pore water. Diffusion processes can also be studied using gel samples immersed in a liquid different from their pore fluid (Hæreid et al. 1995), or gel samples injected with ferrofluid (Salloum et al. 2008). Numerical simulations predict ferrofluid flow and nanoparticle diffusion in cancer cells (Zakariapour et al. 2016). The results of these studies show that ferrofluid can diffuse in the gel pore structure, thus providing potentially useful impregnation methods. Environmental engineering applications include ferrofluid flow driven by magnetic forces (Borglin et al. 2000; Oldenburg et al. 2000). Thus, the potential of magnetic forcing to improve impregnation in MPF studies will be investigated here.

This study investigates a range of impregnation methods and focuses on (1) comparing their impregnation efficiencies for a variety of samples with porosities of 20– > 70%, and (2) improving the assessment of impregnation efficiency and its spatial variation by measuring the MPF of sub-samples of impregnated cores. Results presented here provide guidance on how to improve impregnation in future MPF studies, and assess the consequences of heterogeneous ferrofluid penetration on MPF interpretation. Additionally, the results presented here help estimate more realistic limits of porosity or pore sizes that are captured by MPFs.

## Materials and Methods

### Sample Description

**Fig. 1:**
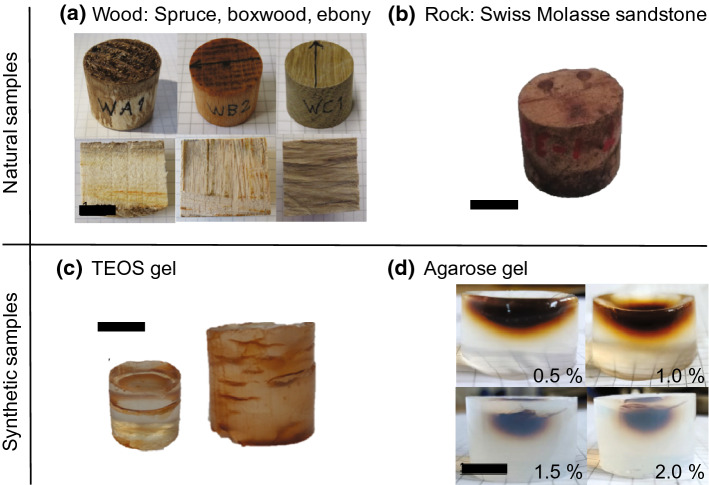
Samples used in this study: **a** Wood: Norway spruce (WA, 70% porosity), European boxwood (WB, 45% porosity), and Macassar ebony (WC, 20% porosity). **b** Rock: Swiss Molasse sandstone from Schüpfheim LU. **c** TEOS gel samples of different sizes, TEOS-1 and TEOS-2, and visible cracks forming preferential impregnation pathways. **d** Agarose gel samples with agarose concentrations of 0.5%, 1%, 1.5%, 2%, and with clearly visible impregnation front. Scale bares indicate 1 cm

**Fig. 2:**
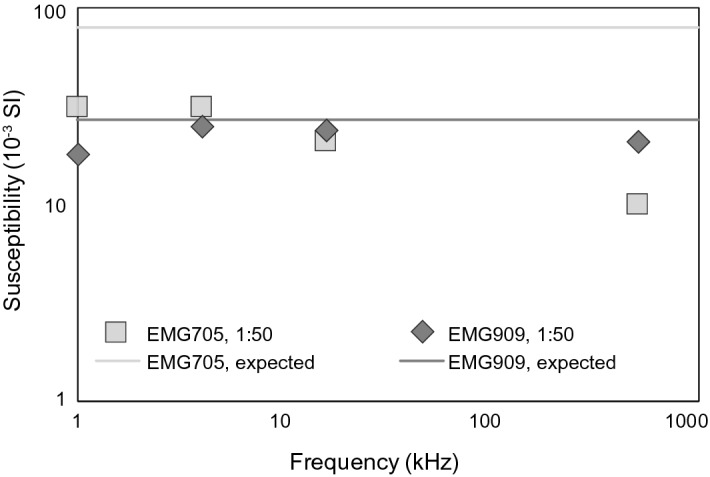
Ferrofluid susceptibility as a function of measurement frequency, compared to expected susceptibility at the same concentration for EMG705 and EMG909

**Fig. 3:**
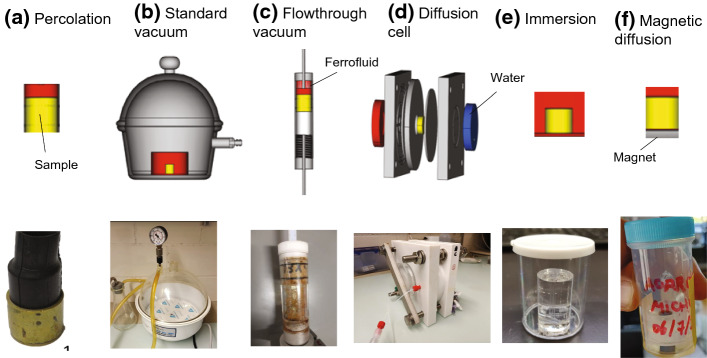
Schematic sketches (top) and photographs (bottom) of investigated impregnation methods: **a** Percolation, **b** standard vacuum, **c** flowthrough vacuum, **d** diffusion cell, **e** immersion, and **f** magnetic diffusion

**Fig. 4:**
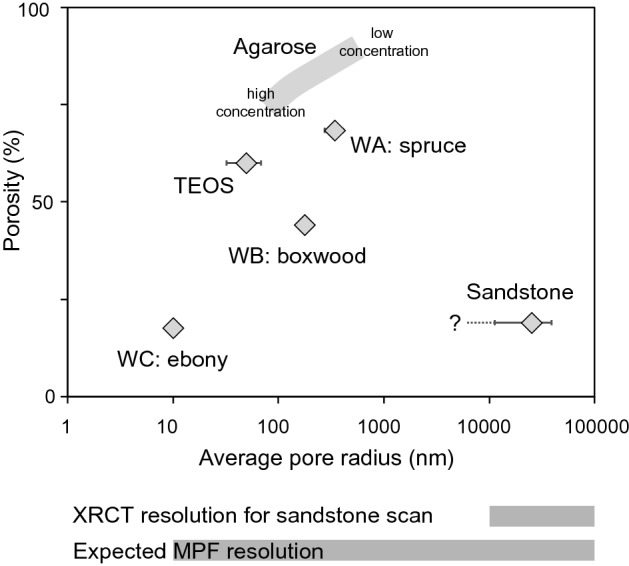
Porosity determined by helium pycnometry for wood and Swiss Molasse sandstone, and porosity values estimated from literature for TEOS and agarose gel. Measurements were done with N_samples_ = 4 and 5 cycles each. The information on the sandstone pore size is limited by the 10 µm resolution of the XRCT measurement. Average pore size values for wood and gels are taken from literature (see text for details)

Investigating the impregnation efficiency and its dependence on pore space properties is crucial in this study; therefore, samples were chosen to cover a wide range of porosities (20 – > 70%), and different pore sizes. The sample collection includes (1) wood cylinders of Norway spruce, European boxwood, and Macassar ebony with porosities ranging from ~ 20% to ~ 70% (Fig. 1a), (2) Swiss Molasse sandstone from Schüpfheim (LU, Switzerland), chosen from a larger collection of rock samples because of its homogeneity, and intermediate porosity of 20% ± 5% which is similar to the porosity of ebony (Fig. 1b), (3) silica gel synthesized from tetraethoxysilane (TEOS), with expected porosity of 50%—60% and pore sizes of 5–30 nm based on the synthesis protocol (Xi et al. 1995) (Fig. 1c), and (4) agarose gel prepared with agarose powder and water at concentrations of 0.5%, 1%, 1.5% and 2% to obtain porosities > 70% and pore sizes ranging from 80 nm (2%) to several 100 nm (0.5%), where both porosity and pore size decrease with increasing concentration (Gu et al. 2004; Narayanan et al. 2006; Pluen et al. 1999) (Fig. 1d). A sample coordinate system was introduced, where *z* is along the cylinder axis, and x chosen along an arbitrary direction normal to it.

Although MPF studies are normally conducted on rocks, wood samples provide some advantages: (1) the wood internal structure is visible and homogeneous, (2) wide ranges of porosity (20% to 70%) and pore sizes (2 nm to 50 μm) are covered by different wood types (Plötze and Niemz 2010), (3) a wide range of wood types is commercially available, and (4) impregnation of wood is often used in construction material science, and their natural anisotropy makes the suitable for this study (Locs et al. 2008; Merk et al. 2014; Oka et al. 2002). Standard cylindrical cores (25 mm diameter, 22 mm length) were cut parallel or at an angle to the visible structure of the wood fibres.

Sandstone from the Swiss Molasse Basin was collected in the Schüpfheim area (LU, Switzerland). The sandstone is classified as fluvial feldspathic litharenite (Von Eynatten 2003). The samples have relatively large pores visible at the X-ray computed tomography scale (~ 10 μm resolution) (Zhou et al. in review), but additional smaller pores are also present. Samples were cut from a single block and drilled to standard-sized cylinders for AMS and MPF analyses. An additional smaller cylinder of 10 mm height and 25 mm diameter was prepared to fit in the diffusion cell.

Synthetic gel samples have the advantage of being uniform in terms of porosity, pore size distribution and matrix composition (SiO_2_). Additionally, their transparency allows for visible investigation of the ferrofluid migration inside the sample, and porosity and pore size can be controlled by adapting the synthesis protocol (Fidalgo et al. 2003; Hæreid et al. 1995; Xi et al. 1995). Tetraethoxysilane gel samples were synthesized in the Geochemistry Laboratory, University of Bern. Samples were prepared with different dimensions (1 cm diameter × 1 cm height (TEOS-1) and 1.5 cm × 1.5 cm (TEOS-2)), to investigate the influence of sample size on gel stability and crack development. TEOS gel 98% reagent (Sigma-Aldrich, Switzerland) was used in combination with distilled water, HCl (7%) and ethanol. The synthesis followed a semi-batch system (Kim and Kim 2002) where two solutions, one consisting of 10 ml TEOS and 1 ml ethanol and the second of 1 ml ethanol, 3 ml water and 0.2 ml HCl, were mixed at room temperature in proportions defined by the target porosity and pore structure. The mixture was then allowed to polymerize for two weeks, and placed in a beaker filled with distilled water for aging and to release ethanol from the pore structure. After that, the samples were ready to be impregnated. Both samples display visible cracks, caused by exposure to the surface, that act as preferential paths for ferrofluid to access the interior of the sample (Fig. 1c). Agarose gel samples were prepared by mixing agarose powder (Sigma Aldrich CH) with near-boiling distilled water to reach target concentrations of 0.5%, 1%, 1.5% and 2%. After heating and mixing in a magnetic stirrer for 30 min, the solution was poured in 50 ml syringes to obtain cylindrical samples of 10 ml volume and with dimensions of 21 mm length × 25 mm diameter. The samples were cooled to room temperature, placed in a plastic container and stored in a fridge to prevent disaggregation during storage and impregnation.

### Initial Sample Characterization

The total porosities of the wood samples and the Swiss Molasse sandstone were determined with a helium pycnometer, model Micromeritics AccuPyc II 1340® in the Geochemical Laboratory at the University of Bern (Switzerland), together with independent bulk volume measurements. Five cycles of helium injections were used to define repeatability and data quality. Results were compared to values published in Plötze and Niemz (2010) for wood, and Chevalier et al. (2010) for Molasse. The TEOS gel samples were not stable enough for porosity measurements, and the pores of the agarose were filled with water. Therefore, porosity and pore sizes were estimated from the synthesis protocol. Alternative methods to measure porosity and pore size distributions, such as mercury intrusion porosimetry or nitrogen adsorption (Giesche 2006; Klobes et al. 1997; Plötze and Niemz 2010; Sing 2001), were not used, as high pressures may damage the samples, and the small samples used for nitrogen adsorption may not be representative.

### Magnetic Anisotropy Measurements

The AMS of the unimpregnated wood samples was measured with the SM150H/L magnetic susceptibility meter (ZH instruments, Czech Republic), while rock samples were measured with the MFK1-FA kappabridge (Agico, Czech Republic). The former was used initially because it covers a larger frequency range. Measurements were taken at three frequencies, 4 kHz, 16 kHz and 512 kHz, and at 80 A/m, the largest field intensity available at all frequencies. Later measurements were done on the MFK1-FA, because of its better data quality and reproducibility, at standard settings of 1 kHz and 200 A/m. Directional susceptibilities were determined following the 15 positions measurement scheme described in Jelinek (1977). Directional susceptibilities were measured 10 times for wood, and 5 times for rock samples, to improve data quality, particularly at frequencies with high instrumental noise, and to assess the significance of the anisotropy (Biedermann et al. 2013). The parameter *R*_*1*_ evaluates the significance of anisotropy by comparing directional variation against the variability of repeat measurements. For example, for 10 repeated measurements, the anisotropy is considered significant if *R*_*1*_ > 0.4, and masked by experimental noise otherwise. The AMS of the TEOS and agarose gel samples was not measured due to their delicate structure. When significant, AMS was then described by the best-fit susceptibility magnitude ellipsoid, or the corresponding symmetric second-order tensor, whose eigenvalues (*K*_*1*_ ≥ *K*_*2*_ ≥ *K*_*3*_) and eigenvectors reflect the principal susceptibility axes (maximum, intermediate and minimum) and their directions. The uncertainty of principal axis directions is given by the confidence ellipses e12, e23, and e13 (Hext 1963; Jelinek 1977). AMS directional results are shown on lower-hemisphere equal-area stereographic projections with Kamb density contours indicating the clustering of maximum and minimum susceptibility directions in repeat measurements (Vollmer 1995). Contours indicate intervals of 2 standard deviations. Magnetic foliation and lineation were used in addition to fabric orientation to visualize variability between measurements. Anisotropy is further described by the parameters *L* (magnetic lineation), and *F* (magnetic foliation) (Jelinek 1981), defined as *L* = $\frac{{K}_{1}}{{K}_{2}}$ and *F* = $\frac{{K}_{2}}{{K}_{3}}$.

### Impregnation

The samples were impregnated with water- and oil-based ferrofluid, EMG 705 and EMG 909, respectively, from Ferrotec, USA, with nominal susceptibilities of 4.04 and 1.38 (SI). Most samples were impregnated with a concentration of 1:50 volume ratio ferrofluid:diluent, with expected susceptibilities of 7.9*10^–2^ and 2.7*10^–2^ (SI). However, note that the ferrofluid susceptibility is frequency-dependent due to the superparamagnetic particles, so that the effective susceptibilities at measurement conditions are lower than expected (Fig. 2). This is relevant for our study, as higher susceptibilities lead to higher MPF anisotropy (Biedermann 2019; Biedermann et al. 2021; Jones et al. 2006), and because susceptibility-based impregnation efficiency determination requires reliable susceptibility values. Magnetic susceptibility measurements of reference fluid were corrected for self-demagnetization (Clark 2014; Clark and Emerson 1999). A ferrofluid:diluent ratio of 1:10 was used for TEOS gels, and pure ferrofluid for agarose and magnetic diffusion experiments to increase the magnetic gradient (Table 1).

**Table 1:** Overview of impregnation methods and samples

Sample and fluid	Wood WA = Norway spruce WB = European boxwood WC = Macassar ebony		Rock		TEOS	Agarose	
Method	(EMG 705 1:50)	(EMG 909 1:50)	(EMG 705 1:50)	(EMG 909 1:50)	(EMG 705 1:10)	(EMG 705 PURE)	(EMG 909 PURE)
Percolation	WA1 (Porosity 72%)	WA2 (Porosity 69%)	D1264Y2 (Porosity 19%)	D1253Z2 (Porosity 20%)	Impossible due to fragile gel structure	AG0.5 perc (Concentration 0.5%)	AG_oil0.5 perc (Concentration 0.5%)
	WB1 (Porosity 44%)	WB2 (Porosity 44%)				AG1 perc (Concentration 1%)	AG_oil1 perc (Concentration 1%)
	WC1 (Porosity 19%)	WC2 (Porosity 16%)				AG1.5 perc (Concentration 1.5%)	AG_oil1.5 perc (Concentration 1.5%)
						AG2 perc (Concentration 2%)	AG_oil2 perc (Concentration 2%)
Standard vacuum	Water boiled off before the target vacuum pressure was reached	WA3 (Porosity 66%) WB3 (Porosity 43%) WC3 (Porosity 19%)	Water boiled off before the target vacuum pressure was reached	D1255Z1 (Porosity 18%)	Impossible due to fragile gel structure	Impossible due to fragile gel structure when exposed to vacuum	
Flowthrough vacuum	WA4 (Porosity 65%)	Because oil-based fluid is more affected by particle aggregation, flowthrough was tested with water-based fluid only	D1262X (Porosity 19%)	Because oil-based fluid is more affected by particle aggregation, flowthrough was tested with water-based fluid only	Impossible due to fragile gel structure	Impossible due to fragile gel structure when exposed to vacuum	
	WB4 (Porosity 44%)						
	WC4 (Porosity 17%)						
Diffusion cell	WAdiff (Porosity 67%)	Oil-based ferrofluid is not suitable for long term experiment due to its tendency for particle aggregation	BE1 (Porosity 19%)	Oil-based ferrofluid is not suitable for long term experiment due to its tendency for particle aggregation	Impossible due to fragile gel structure	Impossible because gel structure does not resist long term experiments	
Immersion	Vacuum is a more efficient and fast alternative for these samples (immersion is used for fragile samples)	TEOS-1 (Porosity 50–60%) 1 cm x 1 cm	Impossible because gel structure does not resist long term experiments				
		TEOS-2 (Porosity 50–60%) 1.5 cm × 1.5 cm					
Magnetic diffusion	WA-mag (Porosity 67%)	Impossible because magnetic forcing alone is not fast enough, and particles aggregate over time	BE-mag (Porosity 18%)	Impossible because magnetic forcing alone is not fast enough, and particles aggregate over time	Impossible due to fragile gel structure	AG0.5 mag (Concentration 0.5%)	AG_oil0.5 mag (Concentration 0.5%)
						AG1 mag (Concentration 1%)	AG_oil1 mag (Concentration 1%)
						AG1.5 mag (Concentration 1.5%)	AG_oil1.5 mag (Concentration 1.5%)
						AG2 mag (Concentration 2%)	AG_oil2 mag (Concentration 2%)

The following impregnation methods were tested (Fig. 3; Table 1):

1. Percolation (Fig. 3a): The cylindrical sample was wrapped in a membrane and the ferrofluid was poured on top of the sample. After letting the fluid percolate through the sample for 24 h, the24 cylinder was removed to measure the MPF.
2. Standard vacuum impregnation (Fig. 3b): After drying in an oven, the sample was placed in a vacuum chamber to evacuate the pores, and subsequently, the ferrofluid was supplied for 24 h at 50 kPa. After this, sample was removed to measure the MPF.
3. Flowthrough vacuum impregnation (Fig. 3c): The dry sample was fit in a plastic tube, the ferrofluid was poured on top of the sample, and at the same time a vacuum was applied at the bottom of the sample. A similar method was used by Pfleiderer and Halls (1990), and is included here for its convenient combination of vacuum and directional forcing to improve the impregnation efficiency.
4. Diffusion cell (Fig. 3d): This setup is based on diffusive equilibration of water and ferrofluid in two reservoirs located at opposite sides of a sample, by ferrofluid moving through the sample, and was adapted from Mazurek and Rufer (2018). Diffusion progress was monitored by removing fluid from the water reservoir at regular intervals, measuring its susceptibility and comparing it with the expected equilibrium value. The equilibrium value *K*_eq_ was calculated as the volume average of water, pore fluid, and ferrofluid susceptibilities. While the ratio of observed to equilibrium susceptibility *K/K*_eq_ is not a direct description of impregnation efficiency, it provides information on the progress of the diffusion.
5. Immersion (Fig. 3e): This method was only used for the TEOS samples, as they were not stable enough for the percolation or diffusion methods used on the other samples. The sample was placed in a plastic container filled with water and ferrofluid. As the ferrofluid diffuses from the liquid into the pore space and water is expelled from the pores, the susceptibility of the sample increases while that of the surrounding fluid decreases. Therefore, both susceptibilities were measured daily until the gel structure deceased. The procedure for measuring TEOS gel is delicate due to its fragile structure, and the gel needs to be immersed in fluid also during measurement, to avoid structure shrinkage and fracture development. Thus, after removing the gel from the ferrofluid, its surface was cleaned, and it was placed in a holder filled with water. Repeat measurements were conducted at 4 kHz, 16 kHz, 512 kHz and 80 A/m field intensity. Similar to the diffusion cell impregnation, the change in the sample and fluid susceptibility is calculated as ratio *K/K*_eq_.
6. Magnetic diffusion (Fig. 3f): Magnetic diffusion takes advantage of magnetic gradients to control the motion of ferrofluid (Borglin et al. 2000), and magnetic impregnation experiments were conducted without applying any additional vacuum or pressure force, on wood, rock and agarose samples. To visualize the effect of the magnetic forcing, sets of sister samples were prepared from agarose gel, supplying 10 μl pure EMG705 ferrofluid on top. While the fluid was percolating through the pores without forcing in one sample, the second sample was placed on a cylindrical permanent magnet (magnetic force of 29 N) to accelerate the diffusion. After 14 days, samples were cut in slices normal to the cylinder axis and the magnetic susceptibility of each slice was measured. Additionally, the progress of the ferrofluid front was observed to determine the distribution of the magnetic fluid within the sample. In addition, wood sample WA and one rock sample from the Swiss Molasse were used for this experiment.

### MPF Measurements

Magnetic pore fabrics were measured in the same way as the AMS. To distinguish AMS and MPF results, sub-scripts ‘dry’ and ‘imp’ are used for parameters obtained before and after impregnation. MPF measurements were conducted within a few days after impregnation, to avoid any artefacts that may arise from any changes in ferrofluid properties over time.

### Determination of Impregnation Efficiency

The impregnation efficiency for the percolation, vacuum and flowthrough was determined based on the change in weight and susceptibility during impregnation, analogous to previous studies (e.g. Parés et al. 2016). Mass impregnation efficiency is defined here as

$${\text{I.E}}{._{{\text{mass}}}} = \;\frac{{\left( {{m_{{\text{imp}}}} - \;{m_{{\text{dry}}}}} \right)}}{{{\text{por}}*\;{\rho_f}*{\text{vol}}}}*100\,\left( \% \right),$$

where *m*_imp_ and *m*_dry_ are the masses of the impregnated and dry sample, respectively, ‘por’ the porosity (as a fraction between 0 and 1), *ρ*_*f*_ is the fluid density, and vol is the volume of the sample. Analogously, susceptibility impregnation efficiency is defined as

$${\text{I.E.}}_{\text{susc}} = \frac{(K_{\rm imp} - K_{\rm dry})}{{\text{por}} \ast K_{\rm fluid}} \ast 100(\% ),$$

where *K*_imp_ and *K*_dry_ are the volume-normalized magnetic susceptibilities of the impregnated and dry sample, respectively, and *K*_fluid_ is the effective susceptibility of the fluid. The obtained susceptibility impregnation efficiency should theoretically be equal for all measured frequencies on any given sample. Any differences in impregnation efficiency with frequency were included in the data variability, because they indicate measurement uncertainty.

A new method that is introduced here, is measuring the MPF small cubes and slices cut from the cylindrical samples. In addition to quantifying impregnation efficiency, this sub-sampling allows to investigate the distribution of ferrofluid throughout the sample, and the effect partial impregnation may have on the MPF results. Note that only susceptibility impregnation efficiencies could be obtained on the sub-samples, as their properties prior to impregnation are not known, and unlike *K*_dry_ which is orders of magnitude smaller than *K*_imp_, *m*_dry_ is similar to *m*_imp_ and not negligible. To determine MPFs of the sub-samples, three repeat sets of directional measurements were obtained at 512 kHz and 80 A/m for measurements on the SM150, and 1 kHz and 200 A/m for measurements performed with the MFK1-FA. The higher ferrofluid susceptibility at lower frequency ensures higher MPF anisotropy for a given pore fabric (Biedermann et al. 2021), and therefore we would now recommend 1 kHz frequency. However, this was not known during the initial measurements, and 512 kHz was favoured as it is the frequency with the lowest noise level on the SM150.

The long-term diffusion and immersion experiments allowed the monitoring of impregnation over time, via the ratio *K*/*K*_eq_, but no direct determination of impregnation efficiency during the experiment. Removal of the sample and sub-sampling would have terminated the diffusion and immersion experiments. Sub-samples could not be obtained from the TEOS gel samples, given their fragility. Similarly, cube sub-samples could not be obtained for the agarose samples, but the slightly higher stability of agarose made it possible to cut slices normal to the cylinder axis. The susceptibility and MPF of these were measured at 1 kHz and 200 A/m on the MFK1-FA. Additional information on the distribution of ferrofluid within the TEOS and agarose gels, and its migration through the sample was obtained by visual inspection.

## Results

### Porosity Measurements

Helium porosities of wood and sandstone are similar to the expected values for wood and rock (Chevalier et al. 2010; Plötze and Niemz, 2010), confirming that different samples of the same wood type and the same molasse sandstone display similar porosities. Samples WA have the largest total porosity (67% ± 3%), samples WB display an intermediate porosity of 44% ± 1%, and WC have a low porosity (17% ± 1%). The molasse sandstones possess a porosity of 19% ± 1% (Fig. 4). The uncertainty in helium pycnometry is within 1% of the measured porosity, so that variability between cycles is not shown. Wood is easy to drill and cut, so that the sample volume can be estimated accurately. Slightly larger errors are expected for the rock samples, because they are not perfect cylinders. Because the helium atom diameter is in the range of pycnometers (10^–12^ m), this measurement provides an upper threshold for the fraction of the pore space that can be impregnated with fluid consisting of larger particles, including water or oil molecules, or the magnetic nanoparticles.

### Magnetic Properties Prior to Impregnation

Prior to impregnation, most wood samples show low magnetic susceptibilities ranging from -1.0*10^–5^ to 5.0*10^–6^ (SI) at 512 kHz with data variability ± 5.0*10^–7^ (maximum standard deviation). The dry sandstones show several orders of magnitude higher susceptibility, ranging from 3.0*10^–3^ to 3.5*10^–3^ (SI) at 1 kHz with variability of ± 2.0*10^–6^ for repeat measurements. As expected, there is no correlation with porosity, and the measured susceptibilities are comparable to sedimentary rocks from sandstone and carbonate reservoirs (10^–3^, 10^–4^) (Clark 1997).

**Fig. 5:**
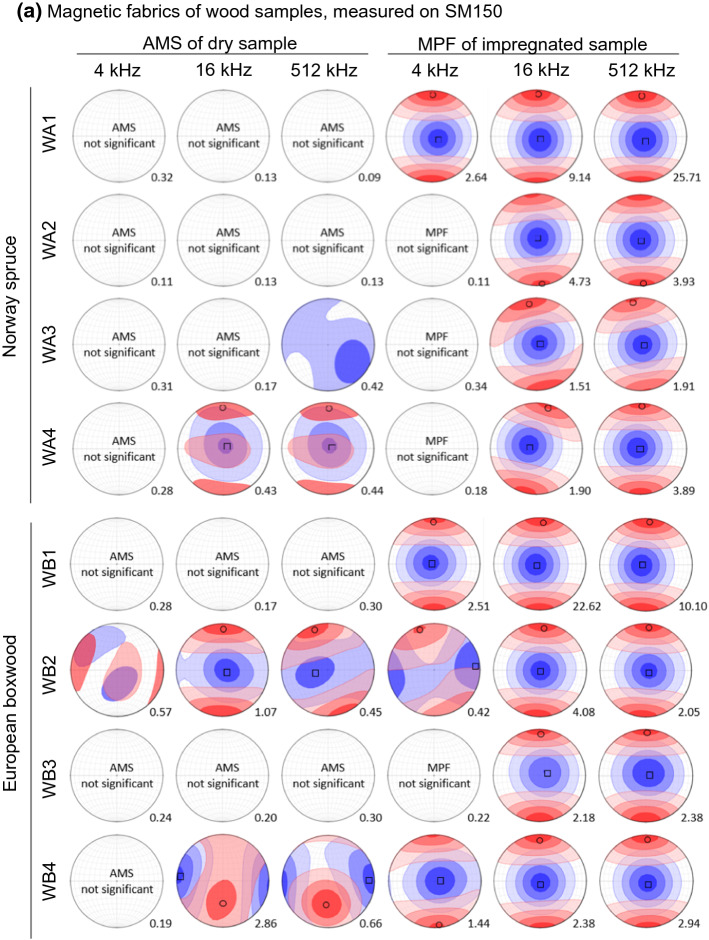

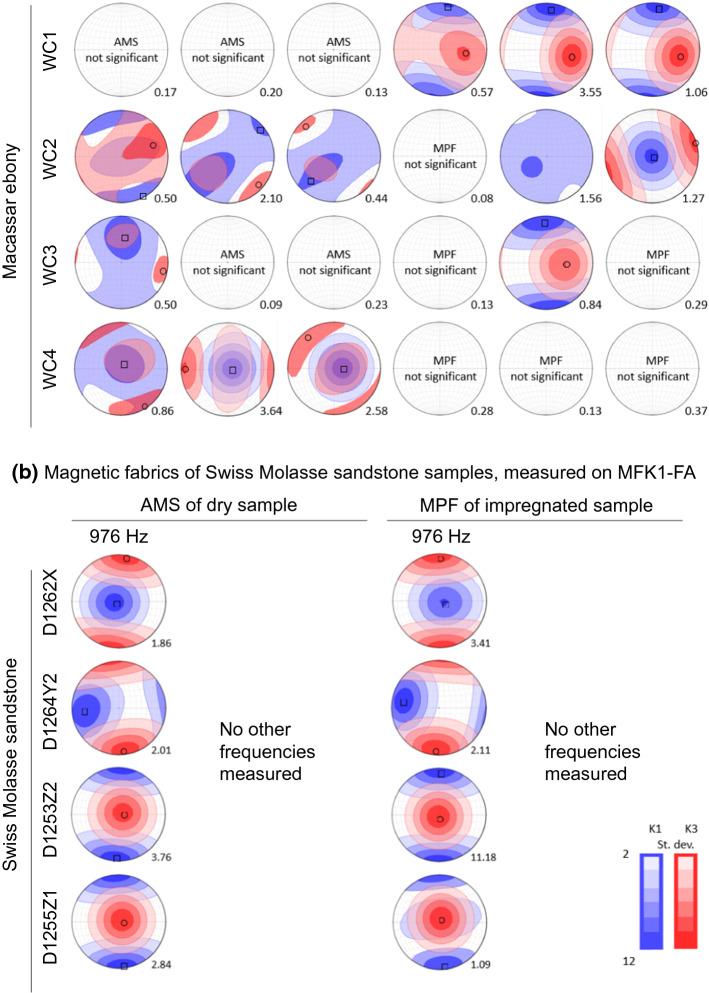
**a** AMS of dry and MPF of impregnated wood samples, at 4, 16 and 512 kHz and field intensity of 80 A/m, measured with the SM150 susceptibility meter. Black circles indicate the mean *K*_*3*_ direction, and black squares the mean *K*_*1*_ direction. Contour plots were derived from individual measurements using a Kamb contour plot. N_measurements_ = 10. N_samples_ = 16 (dry and impregnated). **b** AMS and MPF of impregnated rock samples (Swiss Molasse sandstones). Measurements were conducted on an Agico Kappabridge MFK1-FA at a frequency of 1 kHz and field of 200 A/m with 5 repeated measurements for each position. The number at the bottom right of each plot indicates the *R*_*1*_ value, where thresholds for statistically significant anisotropy are 0.4 for wood (N_measurements_ = 10), and 0.7 for rock samples (N_measurements_ = 5)

Most of the wood samples do not display significant AMS (*R*_*1*_ < 0.4) at 4 kHz, which may be related to a weak anisotropy, or the relatively large noise level of the SM150 instrument. Two out of four WC samples show significant anisotropy, with *K*_*1*_ oriented at 45° to the cylinder axis, parallel to the wood layering (Fig. 1a). All rocks possess significant anisotropy at 1 kHz. The rock samples were drilled from a single block and oriented in the same way, and show similar fabric orientations prior to impregnation, related to a preferred mineral alignment. No frequency-dependence is observed in AMS significance or fabric orientation (Fig. 5).

### Magnetic Pore Fabrics

Impregnated wood and rock samples show significantly higher *R*_*1*_ values compared to the dry samples, and significant anisotropies at all frequencies except 4 kHz measured on the SM150. Only 4 out of the 12 impregnated wood samples display significant anisotropy at 4 kHz, while the same samples have significant anisotropy at larger frequencies (cf. Figure 5). This is contrary to expectation, as MPF anisotropy degrees increase nonlinearly with fluid susceptibility (Biedermann et al. 2021), and the fluid susceptibility is higher at lower measurement frequency (cf. Figure 2). Therefore, the observation that MPFs are only significant at 16 kHz and 512 kHz when measured with the SM150 is most likely attributed to instrumental noise. Rock samples measured on the MFK1 show similar *R*_*1*_ values when dry and impregnated. All measurements indicate significant anisotropy, even if the number of repeat measurements is only *N* = 5 resulting in a threshold value for significant anisotropy of R_1_ = 0.7. This does not necessarily indicate that rocks always have higher anisotropies compared to wood, but can also be related to the lower noise level of the MFK1.

The maximum MPF principal susceptibility of wood samples WA and WB is along the *z* axis, parallel to the orientation of the wood fibres. Also for WC, the maximum MPF susceptibility is aligned with the fibres, which are mostly at 45° to *z*, but show some variability between samples. The MPF of impregnated Swiss Molasse sandstones is co-axial to their AMS, indicating the mineral and pore fabrics have similar orientation (Fig. 5).

### Bulk Impregnation Efficiency

**Fig. 6:**
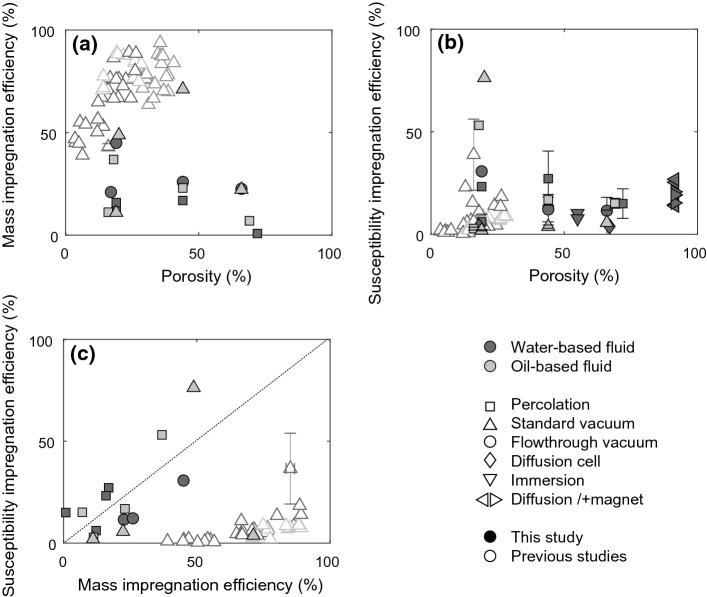
**a** Mass-based impregnation efficiency (I.E._mass_) as a function of porosity, **b** susceptibility-based impregnation efficiency as a function of porosity (I.E._susc_), and **c** correlation between I.E._mass_ and I.E._susc_

Mass impregnation efficiency I.E._mass_ for wood range between 1 – 71%, and the highest values for WA and WB are reached with the standard vacuum impregnation. The lowest I.E._mass_ for wood was observed in WA1, using percolation with water-based fluid. For rock samples, I.E._mass_ ranges between 16 – 49%, which is at the lower end of the range in I.E._mass_ reported previously. Standard and flowthrough vacuum provided the highest I.E._mass_ for our rock samples, 49% and 45%, respectively, and there is no clear trend whether oil- or water-based fluids result in higher I.E._mass_ (Fig. 6a).

Susceptibility-based impregnation efficiency (I.E._susc_) generally differs from I.E._mass_, and covers the range from close to zero to 76%. The highest I.E._susc_ for wood, 27%, was reached by percolation and water-based fluid. Samples WC had I.E._susc_ < 6%, independent of impregnation method. For rock, the maximum value of 76% was obtained by standard vacuum impregnation and oil-based fluid, and diffusion in the diffusion cell was least successful with < 4%. Oil-based fluid results in higher I.E._susc_ compared to water-based fluid. Immersion, diffusion and magnetically assisted diffusion in the gel samples lead to I.E._susc_ of 11 – 27% with water-based fluid, while oil-based fluid was unable to diffuse through the water-filled pores of these samples (Fig. 6b).

If ferrofluid fills the pore space as a homogeneous fluid with constant density and susceptibility, I.E._mass_ and I.E._susc_ determined on the same sample are expected to be equal. This is not always observed (Fig. 6c). For example, wood samples often have a higher I.E._mass_ than I.E._susc_. More pronouncedly, published results on rocks indicate I.E._*mass*_ significantly larger than I.E._susc_. Conversely, for rock samples measured in this study, both estimates of impregnation efficiency are comparable.

### Spatial Variation of Impregnation Efficiency

**Fig. 7:**
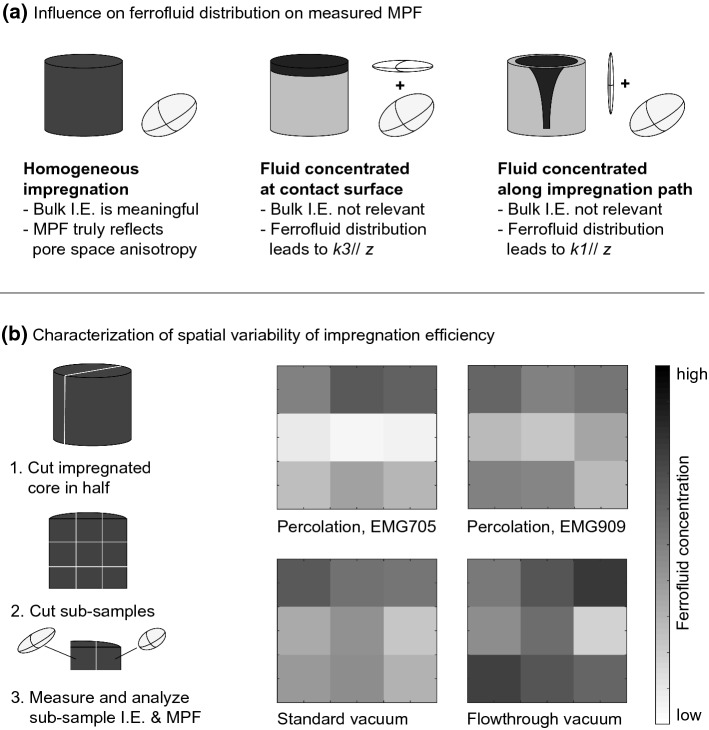
**a** Conceptual sketch showing how incomplete or heterogeneous impregnation may affect the observed MPF. Ellipsoids represent the magnitude of directional susceptibilities, and thus the orientation, degree and shape of the MPF. **b** Workflow to determine the spatial variation of impregnation efficiency and MPF by cutting and measuring sub-samples. Spatial distribution of I.E._susc_ is shown averaged for all samples impregnated with the same method, normalizing all I.E._susc_ values by the highest value achieved in the same sample, to give each sample equal weight

Analysis of the I.E._susc_ of sub-samples shows spatial variation in impregnation efficiency. Spatial variation of impregnation efficiency is relevant as the strong susceptibility of ferrofluid not only causes shape anisotropy in the pores, but the entire impregnated part of the sample generates its own shape anisotropy. The measured MPF is a superposition of both contributions (Fig. 7a). Spatial variation of I.E._susc_ is predominantly controlled by the impregnation method (Fig. 7b). Namely, magnetic nanoparticles aggregated at the sample surface that was in direct contact with the fluid led to higher susceptibility, suggesting I.E._susc_ > 100% in the corresponding sub-samples. Percolation methods lead to low I.E._susc_ in the centre of the sample. Similarly, the standard vacuum impregnation lead to highest I.E._susc_ at the top surface, where the fluid was supplied, but the method is more successful at impregnating the centre of the sample compared to percolation. Although still characterized by spatial variability in I.E._susc_, flowthrough vacuum impregnation appears to provide comparable impregnation efficiency at the centre and surface of the sample. Additional variability was observed between sample groups; e.g., wood samples impregnated with oil-based fluid and standard vacuum impregnation display very low impregnation efficiencies in general, making it hard to investigate spatial variability. Note that the sub-sample susceptibilities added together are lower than the susceptibility of the core from which they were cut. This is due to sample material and nanoparticles removed by the cutting process.

**Fig. 8:**
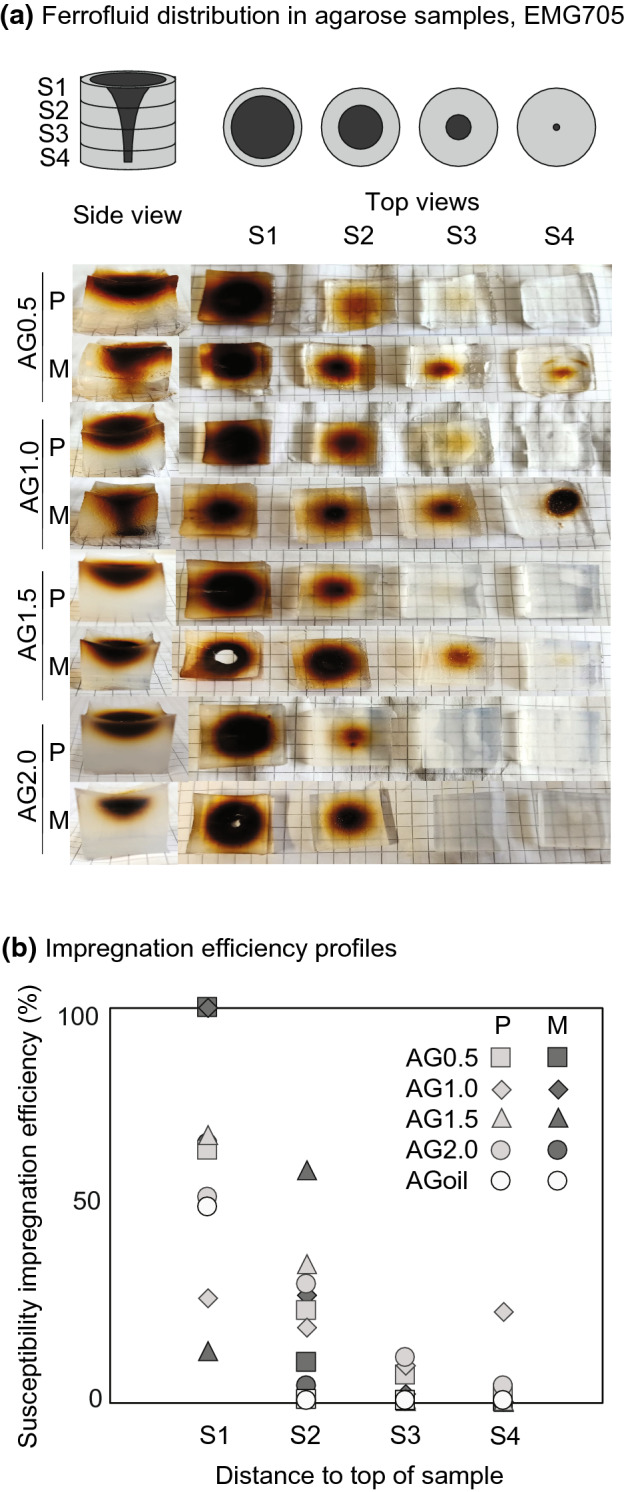
**a** Ferrofluid impregnation in agarose, viewed from the side of the sample, and top views of four slices cut normal to the cylinder axes. The water-based fluid EMG705 had been injected at the top of the sample, and for each concentration one sample was impregnated by letting the fluid percolate and diffuse freely (P), while impregnation in the second sample was assisted by a magnet (M). **b** Profiles of susceptibility-based impregnation efficiency. Pictures of impregnation with oil-based fluids, rock and wood samples are not shown because the fluid stayed at the top of the samples

The transparent gel samples provide a unique opportunity to view the distribution of ferrofluid directly (Fig. 8a). At low agarose concentrations (AG0.5, AG1.0) and with magnetic forcing, a cone-shaped impregnation front is observed. Conversely, at higher concentrations (AG1.5, AG2.0) or in the absence of magnetic forcing the shape of the diffusion front is more rounded. The I.E._susc_ in slices cut normal to the cylinder axis decreases from the top to the bottom of the samples, related to the smaller diameter of the impregnated volume (Fig. 8b). In general, impregnation assisted by magnetic forcing is more successful than pure percolation and diffusion. Impregnation with water-based ferrofluid is successful, while oil-based ferrofluid remains at the top of the sample. Because the pores of agarose are filled with water and oil and water are immiscible, oil-based ferrofluid is not able to migrate through the water-saturated pore space. Similar experiments in rock and wood were hindered by pores becoming dry after short times, resulting in fluid remaining in the upper part of the samples.

### Variation of MPF Between Sub-samples

**Fig. 9:**
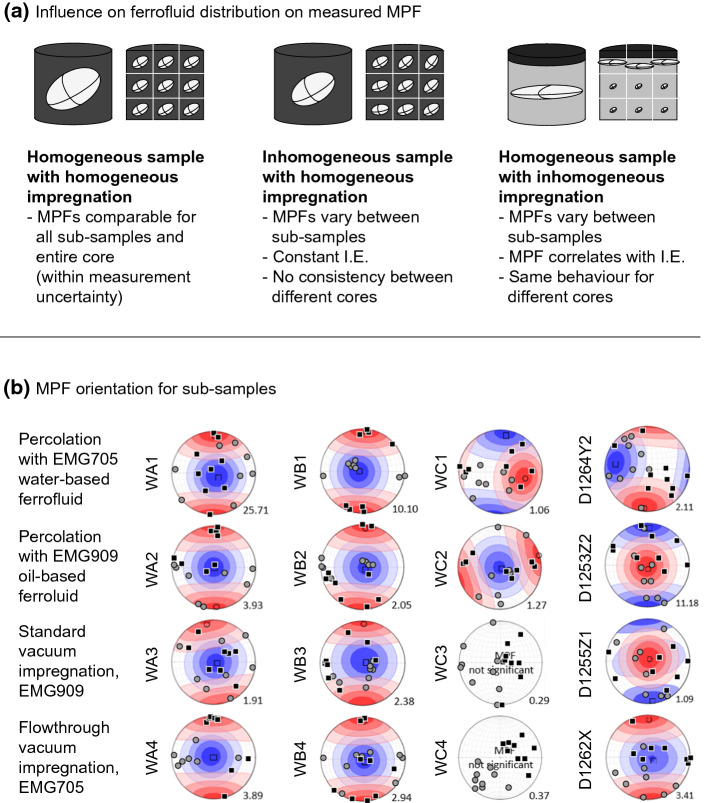
**a** Expected MPFs of sub-samples for homogeneous or inhomogeneous samples, and for homogeneous samples affected by inhomogeneous impregnation. The ellipsoids indicate the magnitude of directional susceptibilities in the bulk core vs the sub-samples. **b** Orientation of MPF maximum (square) and minimum (circle) axes of the sub-samples, compared to the bulk core MPF (color-scale; refer to Fig. 5 for a complete set of bulk core data, and a legend). Wood samples measured at 512 kHz on the SM150, and rock samples at 976 Hz on the MFK1-FA

The MPFs were compared between the sub-samples and bulk cores for all wood and rock datasets with significant anisotropies, to evaluate whether they vary with position in the sample or with impregnation efficiency. If the MPFs are statistically indistinguishable, the sample is homogeneous, and any differences in impregnation efficiency between sub-samples do not affect the MPF result. Conversely, if the sub-sample MPFs are statistically distinct, this implies heterogeneity, or differences in impregnation efficiency affecting the MPF result. Variations in MPF orientation, degree and shape caused by small-scale heterogeneities are expected to be random, unrelated to impregnation efficiency and vary from sample to sample. Heterogeneity could lead to larger variability in samples with bigger pores, because the number of pores captured is less representative. Conversely, if sub-sample MPFs are affected by impregnation-related artefacts, their orientation, shape and degree will show a correlation with impregnation efficiency and with position in the core (Fig. 9a).

When comparing MPFs of cores and sub-samples, it is important to consider that especially anisotropy shape, but also anisotropy degree can show large variability due to noise. For example, the AMS of dry wood showed largely insignificant anisotropies associated with large variability in seemingly high *L* and *F* values, analogous to artificially high *P*-values (*P* = *K*_*1*_/*K*_*3*_) for noisy datasets (Biedermann et al. 2013). Repeat measurements and measurements at multiple frequencies allow estimating the influence of noise. Note that a decrease in *P*, *L* and *F* is expected at higher frequency, where the ferrofluid susceptibility is lower (Biedermann et al. 2021); however, the fabric orientation is independent of ferrofluid susceptibility and measurement frequency. Signal-to-noise ratios are generally lower for the sub-samples compared to the cores, related to their small volume.

Impregnated wood sub-samples were compared to the bulk core at 512 kHz frequency, where the instrumental noise of the SM150 is lowest. Conversely, MPFs of rock samples were compared at 976 Hz, where the anisotropy degree is higher. Sub-sample MPF orientations show large variability, partly due to the higher uncertainty related to the small sample volume. Note that cutting oriented sub-samples was difficult for the rocks, due to their tendency to break or parts chipping of. This introduced additional uncertainty in the sub-sample MPF orientations, making them hard to interpret. For WA1 and WA3, the sub-sample MPF orientations are similar to those of the bulk core, though with large variability. WA2 shows two groups of orientations, and for WA4, most sub-sample *K*_*1*_ directions are at large angles from the bulk core *K*_*1*_. For WB1 and WB2, many of the sub-sample K3 directions are normal to the sample surface that was in contact with the fluid, and may reflect artefacts related to filtered particle aggregates at the surface. The other samples show variable MPF orientations. For the rock samples, D1262X shows the best agreement between bulk core and sub-sample MPF orientation (Fig. 9b).

No clear correlations between spatial variability and ferrofluid type or impregnation method were universally observed. This is reassuring, as it indicates that none of the investigated methods introduces artefacts that consistently affect all samples.

### Time-Evolution of Impregnation

**Fig. 10:**
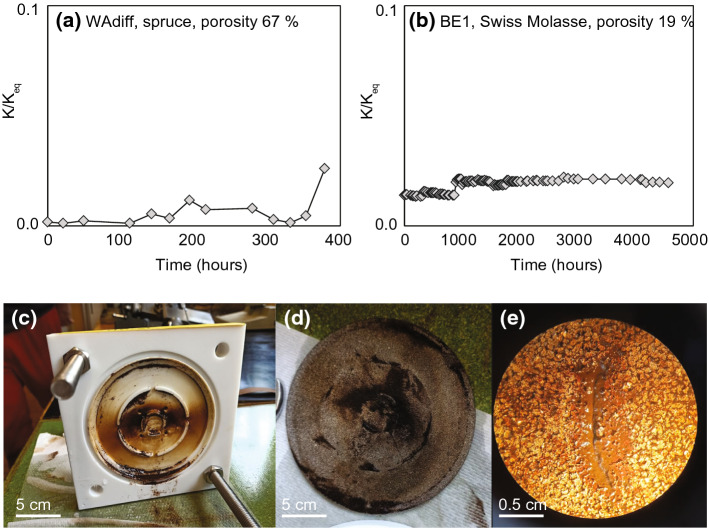
**a** Susceptibility in the receiving reservoir of the diffusion cell for wood sample WAdiff (**a**), and Swiss Molasse sandstone BE1 **b** over time, compared to expected equilibrium susceptibility. **c** Inner part of impregnation cell after disassembling the experiment, **d** ferrofluid particle aggregation on the titanium disc placed between the fluid reservoir and the sample, and **e** reflected light optical microscope image of the particle aggregation on the titanium disc

Impregnation is a time-dependent process, as illustrated by the evolution of measured to equilibrium susceptibility (*K*/*K*_*eq*_) in the water reservoir during diffusion (Fig. 10). For the wood sample WAdiff, *K*/*K*_*eq*_ remains constant for the first ~ 100 h, followed by a small increase ~ 160 h after the start of the experiment (Fig. 10a). This increase is interpreted as the first ferrofluid nanoparticles arriving at the receiving reservoir. Similarly, a second increase in *K*/*K*_*eq*_ after 350–400 h is interpreted as more ferrofluid migrating through the sample and entering the receiving reservoir. The diffusion cell experiment of the Swiss Molasse sandstone BE1 shows a small increase in susceptibility right at the start of the experiment, which we attribute to dissolved rock particles entering the water reservoir. A more pronounced increase in *K*/*K*_*eq*_ in the receiving reservoir is observed ~ 1000 h after the experiment started (Fig. 10b). This indicates that some ferrofluid particles migrated through the sample. After this initial increase, *K*/*K*_*eq*_ in the receiving reservoir decreases again, suggesting that particle migration is not steady, or that the fluid in the reservoir is not homogeneously mixed, i.e., magnetic nanoparticles may settle to the bottom of the reservoir over time. None of the experiments reached the expected equilibrium susceptibility before they had to be discontinued due to sample deterioration. One possible reason could be particle aggregation and filtering in the ferrofluid reservoir, effectively blocking the migration of further nanoparticles through the sample. This is supported by patches of filtered particles observed on the titanium disc that separated the ferrofluid reservoir from the sample after disassembling the diffusion cell (Fig. 10c). The average pore size of the titanium disc is 50 μm, which should be large enough to let the 10 nm particles pass through the pores. However, electrostatic or magnetostatic forces between the magnetic nanoparticles and the metal disc may have favoured aggregation over the long duration of the experiment. Both samples showed signs of weathering due to the prolonged contact with water, *i.e.* the sandstone became unstable, and some wood fibres dissolved in the water, and their MPFs or sub-sample properties could not be analysed.

**Fig. 11:**
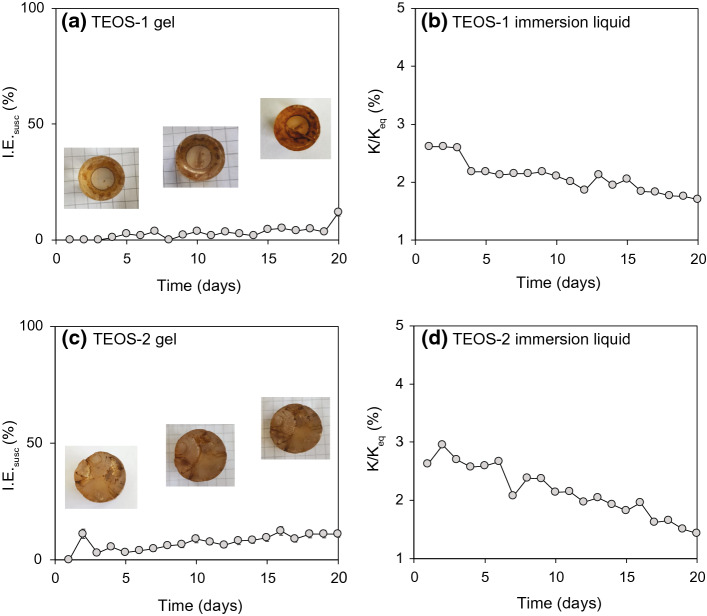
Time-evolution of the susceptibility of the gel samples and surrounding fluid in the immersion experiment. **a** TEOS-1 (cylinder with diameter 1 cm x length 1 cm); **b** ferrofluid where TEOS-1 was immersed; **c** TEOS-2 (cylinder with diameter 1.5 cm x length 1.5 cm; **d** ferrofluid where TEOS-2 was immersed. Ferrofluid uptake by the gel leads to an increase in susceptibility of the gel sample, while at the same time pore water expelled from the TEOS gel leads to a decreasing susceptibility in the surrounding fluid

A direct comparison between the evolution of *K*/*K*_*eq*_ and a visual characterization of the time-evolution of impregnation was possible based on the immersion of the TEOS samples. Because the sample could be measured throughout the experiment, the evolution of the impregnation process is shown as both I.E._susc_ of the sample as a function of time, and *K/K*_*eq*_ of the fluid the sample was immersed in (Fig. 11). The I.E._susc_ of the TEOS gel shows a slowly increasing trend, while the fluid *K/K*_*eq*_ shows a slow decrease, reflecting ferrofluid diffusing into the pores, and pore water being expelled into the surrounding fluid. The I.E._susc_ of TEOS-2 increased more rapidly than that of TEOS-1, and reached higher values, which may be explained by the larger number of cracks that formed as a result of its larger sample size. During the third week of the experiment, a positive peak in susceptibility was observed together with the development of a large fracture that eventually lead to the collapse of the sample after 20 days. In general, increases in I.E._susc_ are associated with the opening of new fractures, visible during the first few days of the immersion experiment for TEOS-2, and towards the end of the experiment for TEOS-1). Full impregnation was not achieved, either because the process was slow compared to the lifespan of the samples, or because ferrofluid particles aggregated inside the pores and clogged fluid migration paths, preventing the migration of additional ferrofluid.

## Discussion

### Orientation of Magnetic Pore Fabrics

The maximum principal MPF susceptibility (*K*_*1,imp*_) in wood is consistently parallel to the visible orientation of the wood fibres. This confirms observations by Merk et al. (2014), and is independent of wood type, porosity and impregnation method. In particular, the directional impregnation enforced by the flowthrough vacuum method did not create a MPF orientation different from non-directional impregnation methods, e.g., standard vacuum. This suggests that the measured MPF is a true representation of the pore fabric, and the pore fabric did not change during impregnation. In the molasse sandstone samples, the MPF is coaxial to the AMS, indicating that the pore fabric reflects the mineral fabric. The MPF orientation appears independent of impregnation method, making us confident that it images pore fabric, and not impregnation-related artefacts. Because the direction of impregnation does not affect the MPF orientation results, samples can be oriented arbitrarily during the impregnation process, so that no a priori information on the sample and its pore fabric is needed.

### Improving Impregnation Methods and Determination of Impregnation Efficiency

The rock samples investigated here show a large variability of impregnation efficiency, with standard vacuum impregnation reaching the highest efficiency with I.E._susc_ = 76%. Percolation with water-based fluid lead to I.E._susc_ = 23% and 53% with oil-based fluid. Flowthrough vacuum impregnation resulted in an intermediate impregnation efficiency of 31%, but lead to the most homogeneous ferrofluid distribution. The same rock samples show larger I.E._susc_ for samples impregnated with oil-based fluid compared to water-based fluid. This supports previous interpretations that oil-based fluid is more suitable to impregnate rock samples Robion et al. (2014), though further investigations are needed to confirm this is the case for all rocks. Because oil is made of larger molecules and has higher viscosity compared to water, it would be expected that water can access smaller pores. Additional forcing may increase impregnation efficiencies; e.g., Locs et al. (2008) impregnated pine wood under pressure (30, 60 and 125 MPa) and report impregnation efficiencies of 96%, and even > 100% since the pressure enlarges and impregnates small fibres that were not previously detected as pores. However, high pressure injection may be unsuitable to study pore fabrics, as it can destroy the pore structure and generate cracks, preventing the characterization of the real pore structure of the sample. Esteban et al. (2006) impregnated rocks at different pressure and reported a change in MPF orientation; however, they did not determine impregnation efficiency, and it is not clear if the changes in MPF are related to different fabrics of different pore sizes or pressure-induced changes. Additional forcing of the ferrofluid migration that has little effect on the pore space itself, is magnetic forcing (Borglin et al. 2000). Comparing the migration of ferrofluid through the sample between percolation and diffusion assisted by magnetic forcing and without additional forcing suggests that future MPF studies will benefit from combining gravitational or pressure forces with magnetic gradients to increase impregnation efficiency, and control the fluid migration path.

Mass- and susceptibility-based estimates of impregnation efficiency differ, because it is the carrier liquid of the colloid that mainly contributes to mass changes, while the magnetic nanoparticles themselves define the susceptibility increase. Thus, I.E._susc_ is most suitable to describe how many nanoparticles entered the pore space. The impregnation efficiencies reported here are at the lower end or below the threshold of I.E._*mass*_ reported elsewhere, while our I.E._susc_ is similar to or larger than reported elsewhere (Almqvist et al. 2011; Nabawy et al. 2009; Parés et al. 2016; Robion et al. 2014). While previous studies systematically reported I.E._*mass*_ >  > I.E._susc_, our results show smaller deviations (cf. Fig. 6c). This is because rather than estimating the expected susceptibility from the nominal fluid susceptibility, this study takes the frequency-dependence of ferrofluid susceptibility into account. For some samples, especially wood impregnated by percolation methods I.E._*mass*_ is still larger than I.E._susc_, and this indicates that some filtering occurred, i.e., the carrier fluid entered smaller pores, but the nanoparticles did not.

None of the experiments proposed here achieved impregnation efficiencies close to 100%. Possible explanations include that (1) the porosity determined by helium pycnometry overestimates the porosity accessible by any fluid, i.e., water, oil and ferrofluid; and (2) the ferrofluid suffers from particle aggregation, further decreasing the accessible pore volume. Particle aggregation and sedimentation has been described in particular for oil-based ferrofluid (Biedermann et al. 2021). Unlike for nanoparticles in water-based ferrofluids, no surfactants are applied to particles in oil-based ferrofluids (ferrofluid.ferrotec.com), and this may explain why they are more prone to particle aggregation and sedimentation. In addition to hindering impregnation, particle sedimentation in larger pores may affect MPF orientation, degree and shape, and needs to be investigated further. Particle filtering appeared stronger in wood samples compared to rocks, evident by differences in I.E._*mass*_ vs I.E._susc_, and may have an additional dependence on mineralogy.

### Time Evolution of the Impregnation Process

Impregnation is a process that proceeds over time, and in particular the diffusion cell experiments have shown that long durations are needed to impregnate samples by water-ferrofluid diffusion. The experiments were running for up to six months (4300 h for BE1) before the samples became unstable and experiments had to be discontinued. Even longer times would have been necessary to reach equilibrium, despite the relatively large porosity of 19% for BE1. Mazurek and Rufer (2018) reported an equilibration time for pore-water diffusion of ~ 3000 h. The slower process for ferrofluid is related to the larger particle size, and may be decelerated further by particle aggregation. Because particle aggregation worsens over time, and due to the increased risk of sample deterioration, we do not recommend long-term diffusion or immersion impregnation for MPF studies. One way to accelerate diffusion is by adding magnetic forcing. The magnetic diffusion in the agarose gels was significantly faster than the non-forced diffusion, and resulted in a more homogeneous impregnation throughout the sample.

While large efforts have been made to study empirical correlations between MPFs and pore shapes or other anisotropic properties, or to improve our understanding of how MPFs arise (Benson et al. 2003; Biedermann, 2019, 2020; Biedermann et al. 2021; Hailwood et al. 1999; Hrouda et al. 2000; Jezek and Hrouda 2007; Jones et al. 2006; Louis et al. 2005; Pfleiderer and Halls, 1993, 1994; Robion et al. 2014), relatively little is known about the ferrofluid impregnation process itself. Valuable insights on this process and its evolution over time were obtained here from impregnating transparent TEOS and agarose gel samples. The TEOS experiments illustrate how cracks developed with time control the speed of the ferrofluid impregnation process; the ferrofluid quickly fills the cracks, and then diffuses more slowly into the surrounding pore space. The agarose experiments allow investigation of an additional effect, the influence of the fluid already present in the pores on the time evolution and shape of the impregnation front. Similar experiments could help understand further factors influencing the impregnation process in future studies.

### Sample and Fluid Properties Affecting Impregnation Efficiency

An advantage of the MPF method that has been put forward is its potential ability to capture pores with throats down to 10 – 20 nm (Almqvist et al. 2011; Esteban et al. 2006; Humbert et al. 2012; Parés et al. 2016; Robion et al. 2014). At the same time, the centre of the samples is not always impregnated (Almqvist et al. 2011; Robion et al. 2014), and Robion et al. (2014) state that ‘depending on the pore throat geometry this [10 nm] threshold is probably much higher’. A higher threshold of impregnatable pore throat size is also confirmed by the spatial variation in impregnation efficiency observed here (cf. Figure 7b), differences between I.E._*mass*_ and I.E._susc_ that indicate that the carrier liquid was more successful entering the pore space than the nanoparticles (cf. Figure 6), and the observation of particle aggregation, e.g. in the diffusion cell (cf. Figure 10). In our attempt to better define porosity and size thresholds above which impregnation is likely successful, we have identified additional factors that control impregnation.

Sample properties that may influence the impregnation efficiency include porosity, pore throat size, pore shape, tortuosity and connectivity, wettability (i.e., mineralogy), and pore fluids already present in the samples. Ferrofluid properties that likely affect impregnation outcomes are viscosity, particle size, and whether the particles have a neutral or electrically charged surface. Oil-based fluids have been considered more efficient at impregnating rocks compared to water-based fluid (Robion et al. 2014). Our results show that water-based fluid is more successful in some samples, e.g. wood and agarose gel, while oil-based fluid leads to more efficient impregnation in others, e.g. molasse sandstone, thus highlighting the influence of sample properties. Particle aggregation and filtering is observed mainly for oil-based ferrofluid impregnating wood, as shown by lower I.E._susc_ compared to I.E._*mass*_. This confirms findings by Biedermann et al. (2021), who reported particle aggregation and sedimentation of oil-based ferrofluid inside voids of synthetic samples. Related differences between the nominal 10 nm nanoparticle size and the effective hydrological diameter of clustered particles limits ferrofluid impregnation to larger pores than previously reported.

**Fig. 12:**
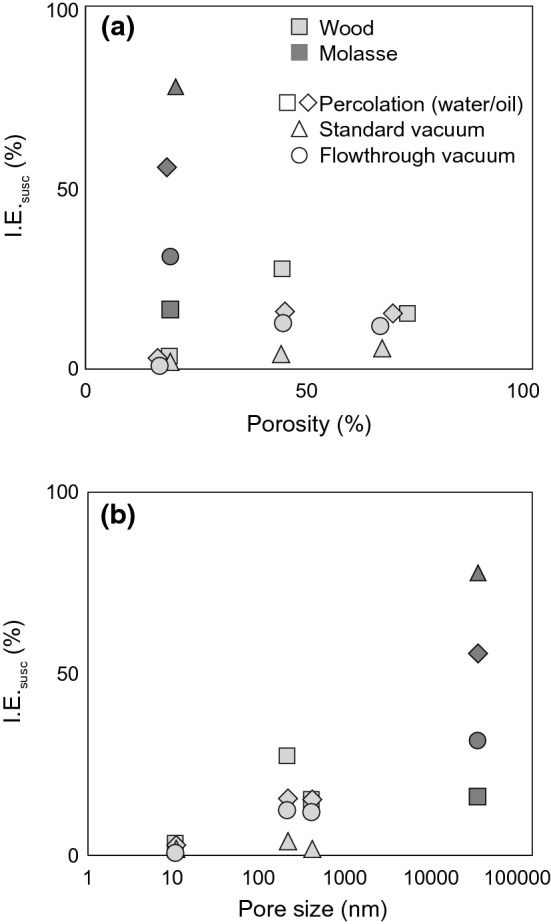
Impregnation efficiency as a function of sample porosity (**a**) and pore size (**b**)

A systematic investigation of all mentioned parameters and their effect on impregnation results is difficult, given the limited ranges in properties of available samples, or co-variation of several properties. Nevertheless, the collection of samples investigated here allows to identify some patterns. No clear correlation was observed between impregnation efficiency and porosity, indicating that porosity is not the controlling factor (Fig. 12a). There is no clear evidence that samples with higher porosity show a more homogenous impregnation efficiency, or a higher impregnation depth. Wood and rock samples with similar porosities (WC and Swiss Molasse, ~ 20% porosity) display different impregnation efficiencies, with rock being more easily and homogeneously impregnated, probably due to their larger pore sizes (10–20 μm for molasse compared to 10—1000 nm for wood). Note that the minimum pore size identified depends on the resolution of the method used, and the 10 μm for molasse is overestimated. The correlation between I.E._susc_ and pore size (Fig. 12b) suggests that size largely controls the impregnation process, with larger pores being impregnated more easily. This is especially evident for samples WC whose pore size is similar to the nanoparticle size, resulting in very low impregnation efficiency. In addition to the control of pore size, we expect lower impregnation efficiencies when long and narrow pore throats are clogged by particles or particle aggregates.

Percolation experiments were performed with water- and oil-based ferrofluid using the same impregnation conditions. These show that the oil-based fluid is more successful impregnating the rock, while for wood, both ferrofluid reach similar impregnation efficiencies in WA, and water-based ferrofluid results in higher impregnation efficiency in WB and WC. This suggests that the material the sample is made of also plays an important role. Wettability describes the ‘preference of a solid to be in contact with one fluid rather than another (Abdallah et al. 2007). The relevance of wettability for MPF studies is two-fold: wettability influences the impregnation process, and it also influences the distribution of ferrofluid inside the pore space when another pore fluid is present. Minerals have different wettability, and quartz and mica have been described as water-wet (Abdallah et al. 2007; Liu and Buckley 1999), while carbonates are reported as water-wet (Abdallah et al. 2007) or oil-wet (Almqvist et al. 2011; Zhang et al. 2006), but wettability also depends on other factors such as pH. Wood is acidic and therefore interacts mainly with basic liquids (Mantanis and Young 1997). The water-based fluid EMG705 has a pH of 8–9, while that of the oil-based fluid EMG909 is not specified (ferrotec.com). While wettability certainly plays a role in impregnation, it cannot explain the observed difference between impregnation results for wood vs rock with water- and oil-based ferrofluid. Another factor is the interaction of the magnetic nanoparticles with the mineral surface by electrostatic forces: silicate surfaces are negatively charged at pH > 2 (Abdallah et al. 2007), and this may hinder impregnation of water-based ferrofluid, where the nanoparticles have an anionic coating to prevent aggregation (ferrotec.com). Conversely, carbonates may be positively charged at pH < 9.5 (Abdallah et al. 2007), which may facilitate the impregnation of water-based ferrofluid with anionic surfactants. Surfactants in general may influence the ferrofluid wetting properties, as they change the surface tension (Latikka et al. 2018). More work will be necessary to investigate these effects in detail, especially concerning the limitations they put on impregnation for specific sample mineralogy.

Water-based ferrofluid is able to migrate through the water-saturated pores of agarose while this is not possible for oil-based ferrofluid, because the latter is not miscible with water. Therefore, in addition to pore size and wettability, the pore fluid already present affects impregnation. This is similar to reservoir rocks whose saturation with hydrocarbons or water affects transport and is important in hydrocarbon exploitation (Abdallah et al. 2007).

To summarize, wood samples are more easily impregnated with water-based ferrofluids while rock samples with oil-based ferrofluids, and pore size is a limiting factor for impregnation efficiency, more so than porosity itself. Sample wettability, the stability of the ferrofluid over time (chemical and physical properties; e.g., evaporation of carrier liquid affects viscosity) and its interaction with the sample (e.g. particle aggregation and filtering) may influence the ability of the fluid to migrate through the sample.

### Influence of Cracks

Cracks affect the mechanical and physical properties of rocks, and may mask any MPF anisotropy due to the pore fabric itself (Humbert et al. 2012). Given the large volume and aspect ratio of cracks compared to pores, it is likely that they outweigh any pore fabric anisotropy. The presence of cracks and fractures also affects the impregnation process itself, shown here by the impregnation of monolithic TEOS gel, where cracks developed after polymerization. These cracks can be viewed as analogies to natural rock samples, where permeability is often controlled by cracks or fractures (Sagar and Runchal 1982). Because the cracks are filled prior to the surrounding pores, their MPF contribution could be corrected for based on the time dependence of MPF results. When measuring the MPF right after impregnation started, and again after a longer time, the difference tensor may indicate the pore fabric. More work is needed to develop such a method and investigate if the different constrictions on nanoparticle motion in pores and cracks may be observed as frequency dependence. One approach for future work would be to measure the MPF of samples with and without cracks and to compare the results to each other. This work would benefit from controlling the crack orientation and geometry.

### Recommendations for Future Work

The ferrofluids used in this study were comparable to other MPF experiments (Almqvist et al. 2011; Benson et al. 2003; Parés et al. 2016; Robion et al. 2014), and some of the results can therefore be related to previously published results. This work confirmed that the central part of the cylindrical samples is difficult to impregnate when using percolation or standard vacuum impregnation methods, resulting in large spatial variability of impregnation efficiency and associated artefacts in MPF orientation, degree and shape (cf Fig. 7). Conversely, vacuum flowthrough impregnation provides more uniform impregnation efficiencies. We have not observed any evidence for artificial fabrics introduced by this directional method, and therefore suggest that more work is done to investigate directionally forced impregnation methods. This could be complemented by testing impregnation along three perpendicular axes, to further evaluate potential preferred directions introduced by the forced impregnation.

Full impregnation of the entire pore space as defined by helium pycnometry is not achieved by any method, because the He atom is smaller than water or oil molecules, or magnetic nanoparticles. Impregnation efficiency is heterogeneous throughout the sample, so that bulk weight or susceptibility changes alone may not be sufficient to determine the impregnation efficiency. Susceptibility-derived impregnation efficiencies on sub-samples provide the most accurate estimate how much ferrofluid entered the pore space, and if there are variations with position in the sample. Care has to be taken when comparing measured susceptibilities of ferrofluid-impregnated samples with the susceptibilities reported in the fluids’ technical specifications, because ferrofluid susceptibility is frequency-dependent (Biedermann et al. 2021). Here, the fluid susceptibility at measurement conditions was determined by direct measurement, taking into account self-demagnetization. Weight changes have to be interpreted with caution, as the largest proportion of the weight is related to the carrier fluid, and not necessarily associated with magnetic nanoparticles in the sample. Differences between I.E._susc_ and I.E._mass_ may indicate particle aggregation, and impregnation behaviour as well as magnetic properties may change as particles agglomerate. The results in this study suggest that impregnation efficiencies < 10% mostly lead to insignificant MPFs. To avoid artefacts associated with filtered particles at the sample surface, we recommend to either compare the MPFs of sub-samples, or to cut off the surface that was in direct contact with the ferrofluid prior to MPF measurements.

Particle aggregation appears to worsen over time, so that long-term impregnation experiments such as diffusion are not suitable. Additionally, despite oil-based ferrofluids being preferred due to higher impregnation efficiency in rocks compared to water-based ferrofluids (Robion et al. 2014), they appear more prone to particle aggregation and associated filtering. Possible particle aggregation is particularly important in samples with pore sizes that can be accessed by 10 nm particles, but not by larger aggregates. Particle aggregation appears to be slower for water-based ferrofluid, which may be associated with their anionic coating, repelling particles from one another. At the same time, the anionic coating may cause electrostatic repulsion with negatively charged silicate surfaces, which may explain why some rocks are more easily impregnated by oil-based ferrofluids, despite their higher viscosity. Therefore, additional considerations will need to be made other than whether the pore throats allow a 10 nm particle to pass mechanically. These include wettability, electrostatic interactions, and potential chemical reactions between ferrofluid and sample. We have been able to partially impregnate samples with pores slightly above 100 nm in size, but not samples with 10 nm pore size, and therefore believe that 100 nm is a more realistic threshold for pores that can be invade by ferrofluid.

Because of the many factors influencing ferrofluid impregnation, no general recommendation that works for all samples can be made. Nevertheless, the results shown here indicate that a ferrofluid with properties similar to the fluid of interest is most suitable. For example, water-based ferrofluids are preferable for groundwater migration studies, while oil-based fluids are likely more suitable for hydrocarbon migration applications. In addition to impregnation properties that depend on pore size, wettability and potential interaction between a ferrofluid and the rock, practical aspects are also important. Water-based ferrofluid is more difficult to handle than oil-based ferrofluid in vacuum impregnation experiments, because the water boils off at room temperature, when the vacuum pressure is ~ 80 kPa or stronger. However, water-based ferrofluid can be used as long as the vacuum can be controlled, as was done here for the vacuum flowthrough impregnation.

## Conclusions

Magnetic pore fabrics are a promising and efficient technique to characterize average pore fabrics in rocks and other materials. The major limitation of this method is related to impregnation efficiency, which is predominantly controlled by pore size, wettability and impregnation method. Here, we have investigated multiple impregnation methods including percolation, standard vacuum impregnation, flowthrough vacuum impregnation, diffusion cell, immersion, and impregnation assisted by magnetic forcing on a collection of samples that covers porosities between 20 and > 70%, and pore sizes from 10 nm to more than 10 µm. We also defined a protocol to quantify the spatial distribution of impregnation efficiency and associated changes in MPFs throughout the sample.

Highest impregnation efficiencies were achieved with standard vacuum impregnation on Swiss Molasse sandstones. However, flowthrough vacuum impregnation leads to more homogeneous impregnation throughout the sample. Magnetic forcing accelerates the impregnation process. Long term experiments are not suitable due to particle aggregation and filtering effects. Therefore, impregnation methods with directional forcing are preferable, as long as it can be verified that they do not introduce artificial pore fabrics. Wood was more easily impregnated by water-based ferrofluid, while Swiss Molasse sandstone with oil-based ferrofluid. Agarose samples whose pore space was filled with water could be impregnated with water-based, but not oil-based ferrofluid. Thus, the choice of ferrofluid used in a particular MPF study will depend on the sample properties and the intended application.

Impregnation efficiency may vary throughout a sample, and this can be tested by measuring sub-samples. We recommend to calculate the susceptibility-based impregnation efficiency, as this directly relates to the amount of magnetic nanoparticles in the pore space. For this calculation it is essential to determine the effective ferrofluid susceptibility at measurement conditions. Incomplete impregnation may bias the MPF interpretation. Therefore, bulk impregnation efficiency as well as its spatial variation need to be evaluated prior to interpreting MPFs. Methodological improvements have been proposed in this work to reach higher impregnation efficiency and homogeneity, and to evaluate impregnation efficiency throughout the sample.

Studying the progress of impregnation on transparent samples and sub-sampling are helpful techniques to better understand the impregnation process itself. The transparent samples have shown the importance of cracks as preferential impregnation paths. More work with similar techniques will help define which parts of the pore space are invaded by ferrofluid and what MPFs measure, and also provide more details on the influence of sample and pore fluid properties.

## Supplementary Information

Below is the link to the electronic supplementary material.

Supplementary file1 (XLSX 101 KB)

## Data Availability

The data is available from the online Supplementary, and on Zenodo: 10.5281/zenodo.6400741.
